# Andrographolide Induces ROS-Mediated Cytotoxicity, Lipid Peroxidation, and Compromised Cell Integrity in *Saccharomyces cerevisiae*

**DOI:** 10.3390/antiox12091765

**Published:** 2023-09-14

**Authors:** Tanaporn Phetruen, Bloem van Dam, Sittinan Chanarat

**Affiliations:** Laboratory of Molecular Cell Biology, Department of Biochemistry, Center for Excellence in Protein and Enzyme Technology, Faculty of Science, Mahidol University, Bangkok 10400, Thailand

**Keywords:** andrographolide, green chiretta, vacuole fragmentation, ER stress, lipid peroxidation, ROS

## Abstract

Andrographolide, a bioactive compound found in *Andrographis paniculata*, has gained significant attention for its potential therapeutic properties. Despite its promising benefits, the understanding of its side effects and underlying mechanisms remains limited. Here, we investigated the impact of andrographolide in *Saccharomyces cerevisiae* and observed that andrographolide induced cytotoxicity, particularly when oxidative phosphorylation was active. Furthermore, andrographolide affected various cellular processes, including vacuole fragmentation, endoplasmic reticulum stress, lipid droplet accumulation, reactive oxygen species levels, and compromised cell integrity. Moreover, we unexpectedly observed that andrographolide induced the precipitation of biomolecules secreted from yeast cells, adding an additional source of stress. Overall, this study provides insights into the cellular effects and potential mechanisms of andrographolide in yeast, shedding light on its side effects and underlying cytotoxicity pathways.

## 1. Introduction

Andrographolide is a natural bioactive compound that is found in the leaves and stems of the *Andrographis paniculata* plant, commonly known as the “king of bitters” due to its extremely bitter taste. For centuries, it has been widely used in traditional medicine for its potential therapeutic properties to treat various ailments, e.g., fever, infections, digestive disorders, and respiratory infections [1,2,3]. This compound has gained attention from the scientific community because of its potent pharmacological activities. Many studies have shown that it exhibits a wide range of beneficial effects, including anti-inflammatory, antioxidant, antibacterial, antifungal, antiviral, anticancer, and immunomodulatory activities [1,3,4]. Thus, andrographolide has emerged as one of the promising natural compounds for the prevention and treatment of several diseases, i.e., cancers, diabetes, and pathogenic infections. These potential benefits of andrographolide have made it a subject of intense research, and its medical applications are being extensively explored.

Although andrographolide is a natural compound with several potential therapeutic benefits, it may cause some side effects in certain individuals [5,6]. The most common side effects reported with the use of andrographolide include gastrointestinal disorders, skin and subcutaneous tissue disorders, followed by anaphylaxis, general disorders, and abnormal administration site conditions [6]. In some cases, the use of andrographolide may also cause allergic reactions, skin rashes, and breathing difficulties. Though most of its side effects are mild to moderate, it can cause life-threatening or lethal anaphylactic shock in a small number of patients, particularly patients with a history of allergies [6]. It is therefore of importance to be aware of the possible side effects and to use them under the guidance of a healthcare professional. Unfortunately, our current understanding of the underlying mechanism responsible for the side effects of andrographolide remains incomplete. It is surprising to note that even basic research on one of the most widely utilized model organisms, the budding yeast *Saccharomyces cerevisiae*, has not been extensively conducted in this regard [7].

To gain more insight into the mechanism of action of andrographolide side effects, we employed the budding yeast *S. cerevisiae* in our study. Here, we observed that andrographolide caused cytotoxicity, especially when oxidative phosphorylation was active. We also demonstrate the impact of andrographolide on various cellular processes such as vacuole fragmentation, ER stress, lipid droplet accumulation, reactive oxygen species levels, and lipid peroxidation, and ultimately a compromised cell integrity. We finally discuss a model in which andrographolide may cause cellular stress and how the cells response to it in budding yeast.

## 2. Materials and Methods

### 2.1. Yeast Strains, Plasmids, Primers, and Growth Conditions

Yeast strains, plasmids, and primers are listed in Supplementary Tables S1–S3, respectively. The methods for yeast genetic manipulation and growth conditions were employed as previously described [8]. Briefly, yeast strains isogenic to W303 were used and grown in YPD (1% yeast extract, 2% peptone, 2% glucose) and a synthetic complete medium containing either 2% glucose (SD) or 2% glycerol (SG). Construction of deletion mutants was conducted by a PCR-based method with the following thermocycler conditions: 95 °C, 3 min; 30 cycles of [95 °C, 30 s; 54 °C, 30 s; 68 °C, 3 min]; 68 °C, 10 min. The deletion mutants were confirmed by colony PCR [9]. Deletion of all genes was accomplished by replacement with a G418-resistant cassette from pFA6a-*kanMX6* [10]. For fluorescent protein tagging, yeast proteins were tagged by gene replacement using the pop-in/pop-out method to maintain endogenous expression levels [11]. For growth curve analyses, 200 μL of liquid media with or without 1 mg/mL andrographolide (Tokyo Chemical Industry, Tokyo, Japan, >98% HPLC) dissolved in DMSO was added to cells in a 96-well plate, incubated at 30 °C, and the optical density at 600 nm (OD600) was measured using a Tecan Spark 10M microplate reader (Tecan Trading AG, Männedorf, Switzerland). Data were then imported to the Prism program (GraphPad, La Jolla, San Diego, CA, USA) to generate growth curves as described previously [12].

### 2.2. Fluorescence Microscopy

In order to study the effect of andrographolide on specific cellular compartments of yeast cells, fluorescence-fused proteins were employed. A single colony of yeast cells was used to inoculate 3 mL of SG media. The log-phase cultures were treated with 1 mg/mL andrographolide or DMSO, which served as control. After 1 h of treatment, cells were harvested by centrifugation at 6000× *g* for 5 min at 25 °C and resuspended in sterile water. Subsequently, 3 μL of cells was dropped onto agarose pads prepared on a microscopic slide before being subjected to observations using an FV1000 confocal laser scanning microscope (Olympus Life Science, Bangkok, Thailand) [13].

Morphologies of yeast vacuoles were observed by using an FM4-64, which labels the vacuolar membrane [14]. A single colony of yeast cells was cultured overnight in YPD supplemented with 1 µM of FM4-64 (Invitrogen, Waltham, MA, USA). The culture was then inoculated to fresh YPD media and incubated to log-phase before adding 1 mg/mL of andrographolide or DMSO serving as control. After 1 h treatment, cultures were collected, washed once with phosphate-buffered saline (PBS), and visualized using a Cy3 filter under a confocal microscope.

Lipid droplets in yeast were visualized using BODIPY 493/503 staining [15]. Log-phase cells were treated with 1 mg/mL andrographolide, with DMSO serving as control. After 0.5, 1, and 3 h, 1 mL of cells was stained with 1 μg/mL BODIPY 493/503 (Sigma-Aldrich, St. Louis, MO, USA) for 10 min at 30 °C. The cells were then harvested by centrifugation at 6000× *g* for 5 min and washed twice in PBS. For fixing the cells, the suspended culture was mixed with an equal volume of fixation buffer (100 mM phosphate buffer, 2 mM MgCl_2_, 8% formaldehyde, and 0.5% glutaraldehyde) and the mixture was incubated in the dark for 20 min. The fixed cells were then collected by centrifugation at 6000× *g* for 5 min and washed twice in PBS. Subsequently, the cells were visualized using an EGFP channel under a confocal microscope.

The level of superoxide in yeast cells was detected by using dihydroethidium (DHE), which could be oxidized to ethidium by superoxide [16]. Log-phase cells were treated with 1 mg/mL andrographolide, with DMSO serving as control. After 0.5, 1, and 3 h, 1 mL of cells was stained with 50 µM DHE (Merck Millipore, Darmstadt, Germany) for 30 min in the dark. Cells were then harvested and washed once with PBS before visualization using a Rhodamine-RedX filter under a confocal microscope. The membrane integrity of yeast cells was determined using propidium iodide staining [17]. After 1 h of andrographolide treatment with DMSO serving as control, cells were collected by centrifugation at 6000× *g* for 5 min and washed once with sterile water. The cell pellet was then resuspended in sterile water supplemented with 6 μg/mL propidium iodide (Sigma-Aldrich, St. Louis, MO, USA) before being visualized by a confocal microscope.

### 2.3. HAC1 Splicing

*HAC1* mRNA splicing was analyzed using a reported protocol with some modifications [18,19]. A single colony of yeast cells was used to inoculate 5 mL of SG media. The log-phase cultures were subjected to different treatments, including 1 mg/mL andrographolide, with DMSO serving as control, 2.5 mM DTT (Gold biotechnologies, Olivette, MO, USA) as the positive control, and sterile water as the negative control. After a 1 h treatment period, these cells were harvested and the total RNA was extracted using an RNeasy Plant Mini Kit (QIAGEN, Hilden, Germany) according to the manufacturer’s instructions. cDNA was then synthesized from 500 ng of total RNA using a TOPscript™ cDNA Synthesis kit (Enzynomics, Daejeon, Republic of Korea) in a final volume of 10 μL. The reaction products were then amplified with the following thermocycle conditions: 94 °C, 1 min; 25 cycles of [94 °C, 30 s; 54 °C, 30 s; 72 °C 60 s]; 72 °C, 10 min. PCR products were run on 2% agarose gels (1× TAE Buffer) at 100 V for 60 min and visualized on a BluPAD Light Transilluminator (Bio-Helix, New Taipei City, Taiwan).

### 2.4. Intracellular ROS Detection

The ROS level in yeast was determined by an oxidant-sensitive fluorescent probe, 2′,7′-dichlorofluorescein diacetate (DCFH-DA) (Sigma-Aldrich, St. Louis, MO, USA) [20]. A single colony of yeast cells was used to inoculate 100 mL of SG media. Log-phase cultures were treated with 1 mg/mL andrographolide or DMSO serving as control. The culture of cells was then mixed with 25 µM DCFH-DA and incubated for 30 min before harvesting by centrifugation at 6000× *g* for 5 min at 25 °C. Cell pellets were washed once in PBS and resuspended in 0.5 mL PBS. An equal volume of glass beads was added and cells were subsequently disrupted by vortexing. The cell lysate was collected by centrifugation at 12,000× *g* at 4 °C for 10 min. Fluorescence intensities of samples were measured with a Tecan Spark 10M microplate reader (Tecan Trading AG, Männedorf, Switzerland) at an excitation wavelength of 490 nm and an emission wavelength of 524 nm. The fluorescence intensity values were normalized to the protein level in the supernatants.

### 2.5. Thiobarbituric Acid Reactive Substances (TBARS) Assay

Lipid peroxidation in *S. cerevisiae* was quantified by determining the thiobarbituric acid reactive substance (TBARS) malondialdehyde as described previously [21,22]. Briefly, log-phase cultures of yeast cells were treated with 1 mg/mL andrographolide or an equal volume of DMSO serving as control. At 0.5, 1, and 3 h after andrographolide treatment, cells were harvested and washed once with sterile distilled water. The pellets were then resuspended in 150 µL PBS and an equal volume of glass beads was added. Cells were disrupted by vortexing and the supernatant was collected after centrifugation at 2000× *g* for 3 min at 25 °C. Then, 1 mL of TBA reagent (0.5 M hydrochloric acid, 12% trichloroacetic acid and 0.375% thiobarbituric acid; Tokyo Chemical Industry, Tokyo, Japan) was added and incubated at 95 °C for 20 min. After cooling, the absorbance was measured at 535 nm using a Multiskan SkyHigh (Thermo Fisher Scientific, Waltham, MA, USA). The concentration of malondialdehyde in the samples was calculated using 1,1,3,3 tetramethoxypropane (Sigma-Aldrich, St. Louis, MO, USA) as standard, and the result was presented as μM of malonialdehyde (MDA)/mg of protein.

### 2.6. Sterols Quantification (Ergosterol Biosynthesis Assay)

Total intracellular sterols were extracted using a method described previously with slight modification [23,24,25]. Briefly, a single colony of yeast cells was used to inoculate 100 mL of SG media containing 1 mg/mL andrographolide and an equal volume of DMSO serving as control. The cultures were incubated for 16 h at 30 °C with shaking. The stationary phase cells were harvested and washed once with sterile distilled water. Samples were collected for total protein quantification. The pellets were then dissolved in 3 mL of 25% alcoholic potassium hydroxide solution and vortexed for 1 min. After mixing, cells were incubated in an 85 °C water bath for 1 h. Sterols were extracted by adding 1 mL of sterile distilled water and 3 mL of hexane and vortexed vigorously for 3 min. The hexane layer containing total sterols was transferred to the clean tube store at −20 °C. Prior to analysis, the sterol extract was diluted five-fold in 100% ethanol and spectrophotometrically scanned between 200 and 320 nm with a UV-Vis spectrophotometer (Shimadzu UV-2600). The ergosterol content was calculated as a percentage of the total protein of the cells by the following equations:

% 24(28) DHE = [(A230/518) × F]/total protein weight % Ergosterol + % 24(28) DHE = [(A281.5/290) × F]/total protein weight

### 2.7. Zymolyase Assay

The membrane integrity of yeast was analyzed using a Zymolyase assay. The cultures were supplemented with 1 mg/mL andrographolide and DMSO serving as control and incubated for 16 h at 30 °C with shaking. The stationary phase cultures were then harvested by centrifugation at 6000× *g* for 5 min at 25 °C, washed once with sterile distilled water, and resuspended in 50 mM potassium phosphate (pH 7.5) and 10 mM 2-mercaptoethanol. Zymolyase-100T (0.4 units) (Zymo Research, Irvine, CA, USA) was added to cell suspensions and the absorbance at 600 nm was measured using a Multiskan SkyHigh (Thermo Fisher Scientific) every 5 min for 1 h [26,27].

### 2.8. Scanning Electron Microscopy

The morphologies of andrographolide-treated yeast cells were observed by scanning electron microscopy (SEM) [28,29]. Briefly, samples were fixed with 2.5% glutaraldehyde (Electron Microscopy Science, Hatfield, PA, USA) for 24 h and washed twice with PBS. Then, a series of dehydrations in various concentrations of ethanol (50–100%) was performed, followed by drying with a critical point dryer. The dried samples were affixed to stubs using double-side adhesive carbon tape and then coated with gold/palladium using a sputter coater (Quorum Technologies, Lewes, UK). Samples were visualized by a Hitachi SU8010 field emission scanning electron microscope (FE-SEM; Hitachi High-Technologies Corporation, Tokyo, Japan) at an accelerating voltage of 10 kV. Images were obtained under 12,000× *g* magnification.

### 2.9. Preparation of Secreted Biomolecules

The secreted biomolecules from yeast cells were prepared by inoculating a single colony of yeast cells to 100 mL of SG media and incubating for 16 h at 30 °C. The culture was centrifuged at 6000× *g* for 5 min at 25 °C and the supernatant was collected. Then, this medium was filtered through a 0.22 µm filter membrane (Merck Millipore, Darmstadt, Germany) to discard yeast cells. The cultured media and fresh media were then mixed with 1 mg/mL andrographolide and DMSO serving as a control before incubating at 30 °C for 1 h with shaking. The mixtures were centrifuged at 10,000× *g* for 10 min at 25 °C and washed once with sterile distilled water. The resulting pellets were collected and run in 4–20% gradient gel before being analyzed by both Coomassie brilliant blue and silver staining.

### 2.10. Quantification and Statistical Analysis

Statistical analyses were carried out using GraphPad Prism version 8.4.2. For details of particular statistical analyses and statistical significance, refer to the figure legends.

## 3. Results

### 3.1. Andrographolide Exhibits Cytotoxicity in S. cerevisiae with ROS Synergy

**Figure 1:**
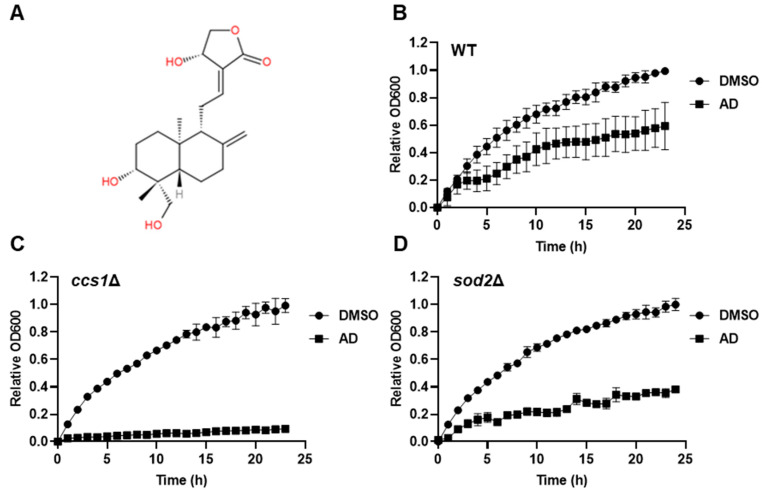
Andrographolide exhibits cytotoxicity in *S. cerevisiae* with ROS synergy. (**A**) Chemical structure of andrographolide. (**B**) Growth curves of wild-type (WT) yeast cells in synthetic minimal media supplemented with 2% glycerol in the presence or absence of andrographolide (AD). DMSO serves as control. Means ± SD (error bars) are shown (*n* = 3). (**C**,**D**) Similar to (**B**), but *ccs1Δ* and *sod2Δ* yeast mutant cells were used, respectively.

To investigate the cytotoxic effect of andrographolide on *S. cerevisiae*, we first treated wild-type yeast cells with andrographolide and monitored the cell growth in synthetic dextrose minimal media by measuring the optical density at 600 nm (OD600). We observed that the andrographolide-treated cells grew significantly slower than the untreated cells, suggesting that to a certain extent andrographolide may cause cell stress and damage and thereby cell growth is inhibited (Figure S1). In yeast *S. cerevisiae*, even under aerobic conditions, the major pathway for energy production is fermentation, in which pyruvate does not enter the mitochondria and is catabolized in the cytoplasm [30]. Since reactive oxygen species (ROS) generated by mitochondrial oxidative phosphorylation during respiration may cause a synergistic effect with andrographolide, we shifted the cells to respiration mode by growing them in a nonfermentable carbon source glycerol and tested again for andrographolide sensitivity [31,32]. Indeed, cells growing in synthetic glycerol minimal media were more sensitive to andrographolide (Figure 1B). Notably, the synergistic impact was even more pronounced in mutant cells *sod2Δ* and *ccs1Δ*, which lack genes involved in the oxidative stress response pathway (Figure 1B,C). These findings suggest that andrographolide-induced stress may be linked to ROS, and the oxidative stress response pathway plays a crucial role in mitigating this stressful condition. Based on these results, we conclude that andrographolide induces cellular damage in *S. cerevisiae*, and this cytotoxic effect is amplified when stress response pathways are compromised, particularly in the presence of activated mitochondrial respiration.

### 3.2. Andrographolide Induces Vacuole Fragmentation, ER Stress, and Lipid Droplet Accumulation in Yeast

**Figure 2:**
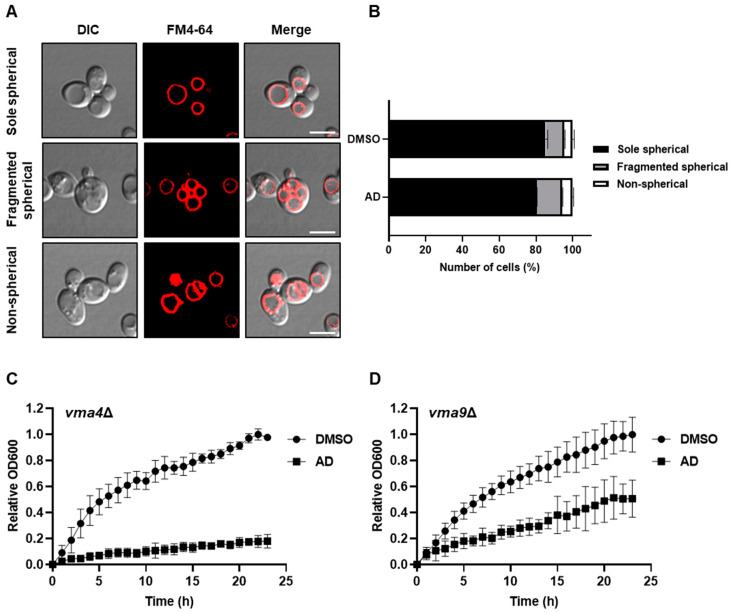
Andrographolide induces vacuole fragmentation. (**A**) Representatives of three vacuole morphologies observed in FM4-64 staining. Scale bar, 5 µm. (**B**) Quantification of different vacuole morphologies in the presence or absence of andrographolide (AD). DMSO serves as control. Means ± SD (error bars) from two replicates with more than 2000 cells per replicate are shown. Statistical analysis was carried out using a two-way ANOVA with Sidak’s comparison between AD and DMSO serving as control conditions (*p* = 0.0009 for sole spherical, *p* = 0.003 for fragmented spherical). (**C**,**D**) Similar to (**B**), but *vma4Δ* and *vma9Δ* yeast mutant cells were used, respectively.

Intrigued by the above findings, we next focused on understanding the specific cellular impairments resulting from andrographolide-induced stress and hypothesized that the administration of andrographolide might lead to stress-related morphological abnormalities in cellular organelles. To this end, we employed fluorescent protein tagging techniques to visualize and define the subcellular distribution of various markers, GFP-Vrg4, Sec7-mCherry, Vps8-mCherry, and Erg11-GFP, which, respectively, represent early and late Golgi, the prevacuolar endosome, and the endoplasmic reticulum (ER) [33]. Our aim was to investigate any morphological changes occurring in these cellular organelles under andrographolide-induced stress. Unfortunately, we did not observe any significant change in morphological abnormalities within those organelles, suggesting that they may not be substantially affected by the andrographolide treatment (Figure S2). Next, we used FM4-64, a lipophilic styryl dye that selectively labels the vacuolar membrane, to observe the morphology of the vacuole. Yeast cells typically possess a single large vacuole, but this organelle can undergo fragmentation in response to various stresses [34,35,36]. Intriguingly, the FM4-64 stain revealed statistically significant morphological changes in the vacuole following exposure to andrographolide. Specifically, we observed an increase in the number of fragmented vacuoles, along with the presence of abnormally formed non-spherical vacuoles (Figure 2A,B). We asked next whether mutations in genes involved in vacuolar function would result in an increased sensitivity to andrographolide treatment. To this end, we generated deletion mutants of *VMA4* and *VMA9* genes, both of which encode crucial subunits of vacuolar H+ ATPase essential for vacuolar acidification [37]. Indeed, when we assessed the sensitivity of these mutants to andrographolide, we observed a notable reduction in the growth of yeast cells (Figure 2C,D). These observations suggest that functional vacuoles play an important role in the tolerance of andrographolide-induced stress.

**Figure 3:**
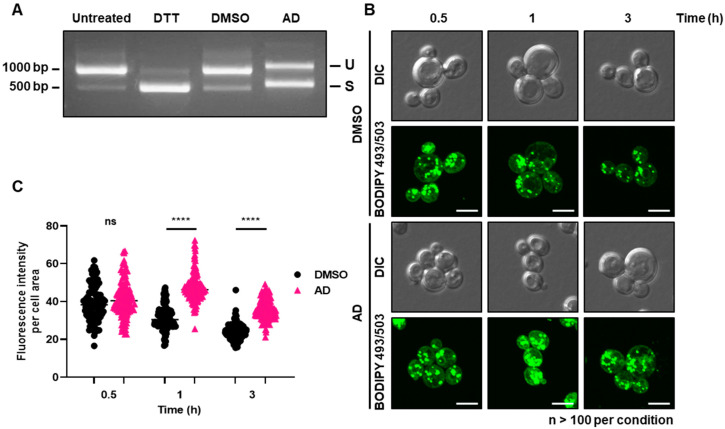
Andrographolide induces ER stress and accumulation of lipid droplets. (**A**) Splicing status of *HAC1* mRNA from yeast cells treated with andrographolide for 1 h and assessed by RT-PCR. DTT and DMSO serve as positive and negative controls, respectively. (**B**) Lipid droplets in cells treated with or without andrographolide for the indicated time period were stained with BODIPY 493/503. Scale bar, 5 µm. (**C**) Quantification of (**B**). Each dot represents the fluorescence intensity per cell area measured by ImageJ 1.53t. Statistical analysis was carried out by two-tailed unpaired t-test with a Mann–Whitney test (****, *p* < 0.0001; ns, not significant).

It has been shown previously that fragmentation of vacuoles in yeast cells can be induced by ER stress [38]; we next hypothesized that the ER itself may be stressed and the ER stress response may be activated upon andrographolide exposure. The ER stress signaling pathway is highly conserved in eukaryotic cells and triggers non-spliceosomal pre-mRNA splicing of *HAC1* mRNA [39,40]. To test this hypothesis, we isolated total RNA from cells treated with or without andrographolide and performed reverse transcription polymerase chain reaction (RT-PCR) to measure *HAC1* mRNA splicing. We used dithiothreitol (DTT)-treated cells as a positive control of ER stress induction [41], which showed the stress-induced splicing of *HAC1* mRNA (Figure 3A). Next, we tested cells exposed to andrographolide, and the RT-PCR showed that *HAC1* splicing seems to be activated under this stress as well (Figure 3A). This result indicates that the exposure to andrographolide most likely induces stresses in both the vacuole and ER of *S. cerevisiae*.

Abnormalities in membrane-bound organelles such as the vacuole and ER have the potential to influence the formation of lipid droplets, which are essential organelles involved in maintaining lipid homeostasis [42,43,44]. Intrigued by the above findings, we next tested whether andrographolide-induced stress could impact the accumulation of lipid droplets. To assess this, we employed BODIPY 493/503, a selective fluorescent stain specifically targeting intracellular lipid droplets [45]. Remarkably, upon exposure to andrographolide-induced stress, we observed a noticeable increase in cellular lipid droplets, starting as early as half an hour after treatment (Figure 3B,C), suggesting that lipid homeostasis, too, is most likely affected by andrographolide. Taken together, we conclude that the treatment of andrographolide leads to the induction of the fragmentation of vacuoles, ER stress, and an increase in lipid droplet formation in yeast.

### 3.3. Andrographolide Induces Lipid Peroxidation and ROS Elevation in Yeast

**Figure 4:**
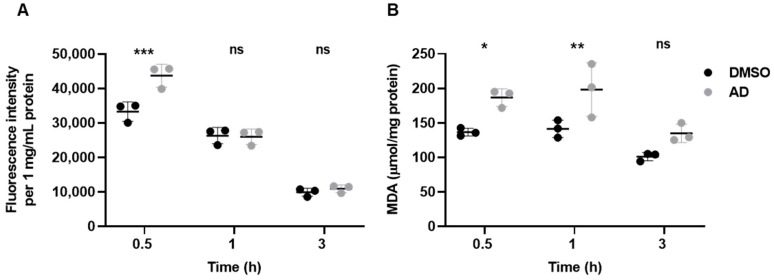
Andrographolide induces lipid peroxidation and ROS elevation. (**A**) Reactive oxidative species (ROS) in cells exposed to andrographolide for the indicated time period were assessed by 2′,7′-dichlorofluorescin diacetate (DCFDA) staining. Each dot represents the fluorescence intensity per 1 mg/mL protein. Error bars indicate the SD of three independent biological replicates. Statistical significance was calculated using a two-way ANOVA with Sidak’s multiple comparisons (***, *p* < 0.001; ns, not significant). (**B**) Similar to (**A**), but ROS-mediated lipid oxidation was assessed by TBARS assay via spectrophotometric detection of its end product malondialdehyde at 535 nm. Each dot represents the amount of calculated MDA in µmol/mg protein unit. Error bars indicate the SD of three independent biological replicates. Statistical significance was calculated using a two-way ANOVA with Sidak’s multiple comparisons (*, *p* < 0.05; **, *p* < 0.01; ns, not significant).

Lipid droplets serve as cytosolic organelles involved in continuous synthesis and turnover, responding to various cellular requirements and stresses [42,43,44]. They possess the unique ability to incorporate excess fatty acids and cholesterol (equivalent to ergosterol in yeast) into their neutral core region as biochemically inert triacylglycerides and steryl esters. Excessive accumulation of reactive free fatty acids in cells can lead to lipotoxicity, resulting in mitochondrial damage, disruption of membrane integrity, and eventual cell death [46,47]. Lipid droplets therefore act as antioxidant organelles that control the storage of fatty acids to reduce toxic lipid peroxidation [46,47]. As shown above, andrographolide treatment resulted in an accumulation of lipid droplets (Figure 3B,C). We next asked whether this phenomenon is a cellular response to an increase in ROS or similar factors, potentially leading to elevated lipid peroxidation in yeast cells. To this end, we treated cells with andrographolide and performed assays to detect superoxide, ROS, and lipid peroxidation using the fluorescent indicators dihydroethidium (DHE) [16,17], dichlorodihydrofluorescein diacetate (DCFH-DA), and thiobarbituric acid reactive substance (TBARS), respectively [20,21,22,48]. While the levels of superoxide remained unchanged (Figure S3), we observed a rapid increase in ROS levels within half an hour of andrographolide treatment, which gradually decreased over time (Figure 4A). This suggests that the generated ROS may not be solely superoxide but could involve other ROS species. Furthermore, we noted an increase in lipid peroxidation, which peaked approximately 0.5 to 1 h after andrographolide exposure, coinciding with a decrease in the ROS signal at 1 h post-treatment (Figure 4B). Interestingly, previous studies on human cancer cell lines have reported that andrographolide induces ferroptosis, a specific form of apoptosis triggered by lipid peroxidation [49,50,51,52]. These results indicate that andrographolide elevates cellular ROS and lipid peroxidation, which can have toxic effects on yeast cells. These findings align with our earlier results demonstrating that yeast mutants deficient in oxidative stress responses exhibited a severe growth phenotype when subjected to andrographolide treatment (Figure 1C).

### 3.4. Andrographolide Compromises Cell Integrity and Affects the Ergosterol-Dependent Membrane Stability

**Figure 5:**
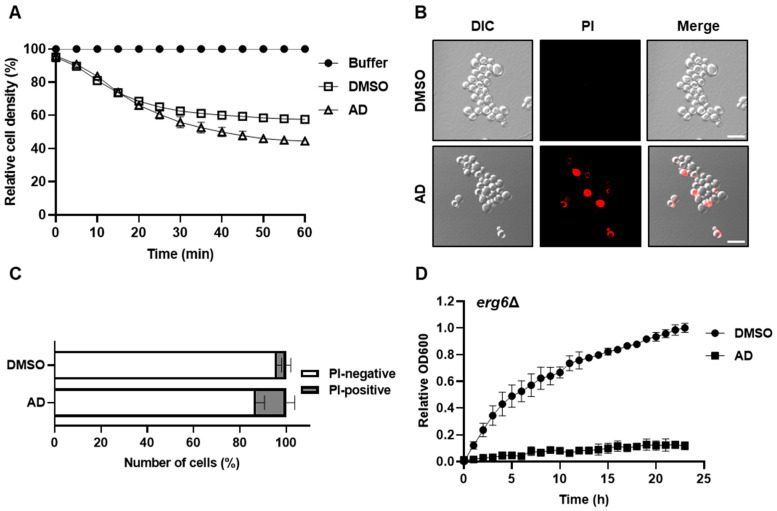
Andrographolide compromises cell integrity and affects ergosterol-dependent membrane stability. (**A**) Zymolyase sensitivity of yeast cells treated with andrographolide. Cells were treated with 0.4 U of Zymolyase and OD600 was observed at the indicated time point. DMSO serves as control. (**B**) Propidium iodide staining of yeast cells treated with andrographolide. DMSO serves as control. Scale bar, 10 µm. (**C**) Quantification of (**B**). Means ± SD (error bars) from three replicates (*n* > 100 per replicate) are shown. Statistical analysis was carried out using a two-way ANOVA with Sidak’s comparison between AD and DMSO serving as control conditions (*p* = 0.017 for PI positive). (**D**) Similar to Figure 1B, but *erg6Δ* mutant cells were used.

Based on the aforementioned data, it appears that andrographolide can disrupt the regulation of specific membrane-bound organelles and induce an imbalance in lipid homeostasis. Building upon this, we hypothesized that andrographolide exposure may also impact the plasma membrane and overall cell integrity. To this end, we employed Zymolyase, a mixture of cell-wall-digesting enzymes, to assess cell lysis rates under conditions with and without andrographolide stress [53]. Notably, cells exposed to andrographolide exhibited faster changes in reduced turbidity following Zymolyase treatment compared to the control cells (Figure 5A). Furthermore, we utilized propidium iodide, a fluorescent indicator that binds to nucleic acids upon membrane damage, to assess cell integrity [17]. Consistent with the Zymolyase assay, the propidium iodide assay revealed a significant increase in the number of cells with membrane damage upon andrographolide treatment (Figure 5B,C). Given that ergosterol, an essential membrane component responsible for maintaining cell membrane integrity and with a selective permeability akin to mammalian cholesterol, plays a crucial role in yeast cell integrity [54,55,56], we investigated if our findings align with this notion. First, we measured the content of cellular ergosterol and observed that the amount of this compound did not considerably change upon andrographolide treatment (Figure S4A,B). However, when we deleted *ERG6*, a gene encoding delta(24)-sterol C-methyltransferase which converts zymosterol to fecosterol by methylating C-24 in the ergosterol biosynthetic pathway, and tested its sensitivity to andrographolide, *erg6Δ* cells were strikingly highly susceptible to the compound (Figure 5D) [55]. From these results, we postulate that while andrographolide may not directly impact ergosterol production, the absence of this compound can compromise cell membrane integrity, rendering cells more susceptible to andrographolide toxicity. Taken together, we conclude that cell integrity is most likely compromised under andrographolide-induced stress.

### 3.5. Andrographolide Induces Sheet-like Structures Impacting Yeast Cells

**Figure 6:**
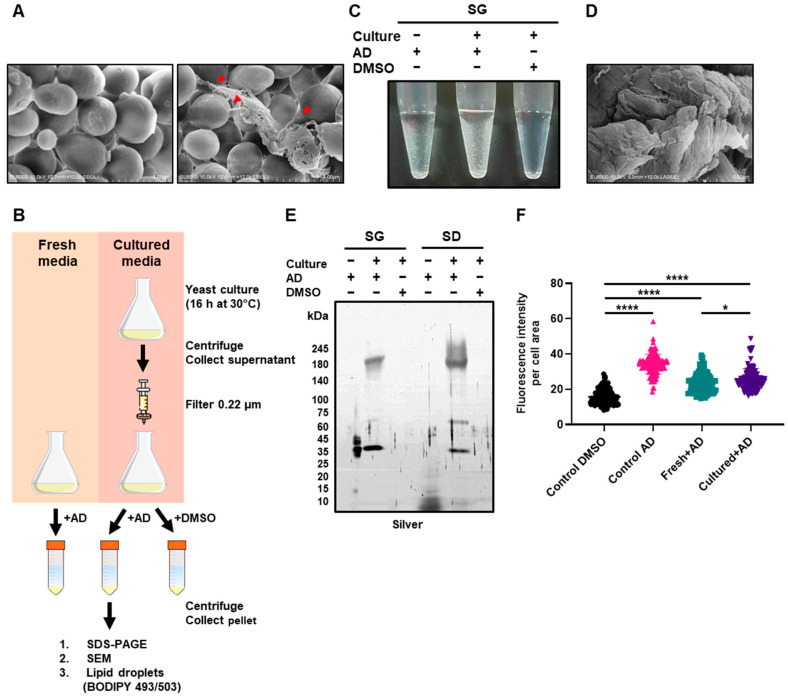
Andrographolide induces sheet-like structures, impacting yeast cells. (**A**) Scanning electron micrographs of yeast cells treated with or without andrographolide. Two representative micrographs are shown. The red arrows indicate sheet-like structures that cover yeast cells. (**B**) A diagram depicting an andrographolide-induced precipitation assay. Either fresh or cultured media were mixed with andrographolide to the final concentration of 1 mg/mL, centrifuged, and washed with sterile distilled water. (**C**) The precipitation in SG media from the experiment represented in (**B**). (**D**) Similar to (**A**), but the pellets formed after mixing andrographolide with cultured media were analyzed. (**E**) The pellets were resuspended in 2× SDS-PAGE loading buffer and subjected to SDS gel electrophoresis followed by silver staining. (**F**) Quantification of fluorescent intensity from BODIPY 493/503 staining. Each dot represents fluorescence intensity per cell area measured by ImageJ (version 1.53t). Statistical analysis was carried out via a two-tailed unpaired *t*-test with a Mann–Whitney test (*, *p* < 0.05; ****, *p* < 0.0001).

Intrigued by the above findings, we next asked whether any cellular surface damage could be observed using scanning electron microscopy (SEM). To this end, we prepared samples of yeast cells treated with andrographolide as well as untreated control cells and subjected them to SEM analysis (Figure 6A). While we did not observe any significant or severe damage on the surface of andrographolide-treated cells, we did notice an atypical feature: the presence of clearly visible connected matrices. By contrast, the untreated control cells exhibited no such structures on their surfaces. The sheet-like structure observed reminded us of an extracellular matrix, a network comprising an array of multidomain macromolecules secreted by the cells which they surround [57]. Given that yeast cells also secrete many proteins of diverse functions—from metabolic enzymes to structural proteins [57]—we next hypothesized that the formation of sheet-like structures might come from secreted proteins of yeast cells which were then reacted with andrographolide in the media culture. To test the hypothesis, we first prepared cultured media by inoculating fresh media with yeast cells and growing them from early log phase to mid/late-log phase. By doing so, yeast cells would secrete proteins and extracellular biomolecules into the media. Next, we added andrographolide to either freshly prepared media or the aforementioned cultured media containing released molecules (Figure 6B). Mixed with andrographolide, the freshly prepared media showed no apparent precipitates and remained transparent. However, strikingly, the cultured media exhibited a clearly visible amount of precipitation, while DMSO serving as control did not show any (Figure 6C and Figure S5A). Being curious about these findings, we next collected the precipitates and subjected them to SEM analysis and observed that they looked very similar to what was observed when cells were treated (Figure 6D), suggesting that what we observed previously might have been the andrographolide-mediated precipitation wrapping around the surface of yeast cells (Figure 6A). Subsequent gel electrophoresis followed by Coomassie Blue and silver staining of the precipitates showed several bands of products only in andrographolide-treated samples but not the DMSO serving as control (Figure 6E and Figure S5B). These results suggest that the andrographolide-induced precipitates likely contain biomacromolecules, including proteins. The formation of precipitates can be attributed to the possibility of andrographolide reacting with nucleophilic molecules such as cysteine residues of proteins and esters through Michael’s reaction [58,59]. It has previously been shown that andrographolide can react with certain proteins, including NF-κB p50 and Keap1 [60,61]. Therefore, we conjecture that andrographolide may covalently react with various biomolecules secreted from yeast cells, causing a reduction in their solubility and resulting in the formation of precipitates.

The presence of sheet-like structures in the andrographolide-treated media raised an intriguing question: were all observed cellular responses primarily a result of free andrographolide entering the cells and inducing such responses, or could the extracellular sheet-like matrix primarily cause these effects from outside the cells? To distinguish these two scenarios, we conducted an experiment using precipitates obtained from the andrographolide-treated media. We first prepared the precipitates by mixing 1 mL of cultured media with andrographolide to achieve the final concentration of 1 mg/mL. After centrifugation, we removed the supernatant which contained the free form of andrographolide. The pellet was then washed twice and resuspended in fresh media. Our aim was to remove any free andrographolide from the pellet, so any effects on yeast cells would originate from the pellet itself. As a positive control, fresh media containing 1 mg/mL of andrographolide was used to treat yeast cells. To assess the cellular effects, we utilized highly sensitive and quantifiable BODIPY 493/503 staining as a measure of stress-induced accumulation of lipid droplets. To our surprise, the andrographolide-induced precipitates were able to induce an increase in lipid droplets compared to the untreated control (Figure 6F). However, the extent of the increase was still significantly lower than that observed with direct andrographolide treatment (Figure 6F). These results suggest that andrographolide may induce cellular stress not only from outside the cells through the extracellular sheet-like matrix but also from within the cells, likely through its penetration into the cells. In summary, we conjecture that cytotoxicity effects from andrographolide might be attributed to both intracellular and extracellular factors.

It is also important to note that, to our knowledge, there is no direct evidence demonstrating either the formation of precipitates between andrographolide and secreted biomolecules or the induction of cytotoxicity from such structures. As most previous studies have primarily focused on investigating the intracellular molecular mechanisms of andrographolide, it would be important to reconsider the potential effects of extracellular structures in future investigations, as they may contribute to the overall impact of andrographolide on cellular systems.

## 4. Discussion

Andrographolide, a natural bioactive compound derived primarily from the plant *Andrographis paniculata*, is widely used in traditional medicine, especially in Asia, to treat a variety of conditions, including the common cold, fever, and digestive disorders. It is recognized for its anti-inflammatory, antiviral, antioxidant, and immunomodulatory properties. However, it is important to acknowledge that andrographolide can also have side effects, ranging from mild to potentially life-threatening. While the molecular mechanisms of action of andrographolide have been studied in specific cells and organisms, its overall effects, especially in budding yeast, remain incompletely understood.

**Figure 7:**
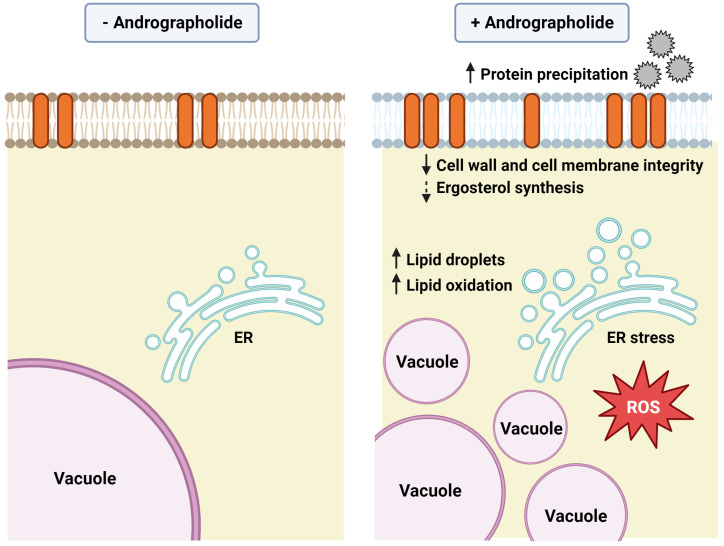
Model of andrographolide’s mechanism of action in budding yeast *S. cerevisiae*. (The figure was created at BioRender.com).

Here, we observed that andrographolide exhibited cytotoxic effects in yeast, particularly when aerobic respiration and oxidative phosphorylation were active. While most organelles appeared normal, vacuoles showed increased fragmentation under andrographolide-induced stress. Furthermore, andrographolide triggered the activation of the ER stress response pathway, an accumulation of lipid droplets, elevated levels of ROS, and increased lipid peroxidation. As a result, cell integrity was likely compromised under andrographolide treatment. Remarkably, we also discovered that andrographolide induced the precipitation of proteins secreted by yeast cells, adding an additional source of extracellular stress. This observation highlights the potential contribution of extracellular factors to the overall effects of andrographolide (Figure 7).

It is important to note that there is some conflicting evidence about the effects of andrographolide on ROS. Some studies, including our own work with yeast, have shown that andrographolide can increase ROS production in cells. However, other studies have shown that andrographolide can reduce ROS production. Thus, how can andrographolide have both antioxidant and pro-oxidant effects? We believe that the effects of andrographolide depend on the dose, the type of cell, and the specific conditions tested. As andrographolide is known to act as an electrophilic compound and interact with the nucleophilic Cys of protein targets or biological thiols via Michael addition at the Δ^12(13)^ exocyclic double bond, it is tempting to speculate that the broad effects of andrographolide are caused by protein targets that are expressed in the cells and their functions or pathways under particular conditions. As the chemical reactions of andrographolide can be broad, so too are their effects, including antioxidant and pro-oxidant properties. From our findings, it would be possible that the formation of andrographolide adducts with proteins and biological thiols may momentarily cause oxidative stress. Reacting to this, cells could recognize this stress and activate their stress response systems. Over time, these cells might then generate specific proteins and enzymes that aid in reducing ROS, thereby decreasing their levels. This may suggest the reasons why andrographolide could exhibit both beneficial and detrimental effects, as observed in different studies. More research is definitely needed to fully understand the contradictory effects of andrographolide on ROS production and oxidative stress. However, our current understanding suggests that the effects of andrographolide are complex and depend on a variety of factors.

## 5. Conclusions

In summary, our study provides insights into the cytotoxicity of andrographolide in yeast. We demonstrate its impact on various cellular processes, including vacuole fragmentation, ER stress, lipid droplet accumulation, ROS levels, lipid peroxidation, and precipitation of secreted biomolecules. These findings in budding yeast contribute to a better understanding of the broader effects of andrographolide, shedding light on potential implications for therapeutic applications.

## Data Availability

No new datasets were created.

## Supplementary Materials

The following supporting information can be downloaded at: https://www.mdpi.com/article/10.3390/antiox12091765/s1, Table S1: strains used in this study, Table S2: plasmids used in this study, Table S3: primers used in this study, Figure S1: andrographolide exhibits cytotoxicity in *S. cerevisiae* with ROS synergy, Figure S2: no significant change in morphological abnormalities of organelles was observed in cells treated with andrographolide, Figure S3: levels of superoxide remained unchanged upon andrographolide treatment, Figure S4: levels of ergosterol remained unchanged upon andrographolide treatment, Figure S5: andrographolide increases precipitation in cultured media. Reference [62] is cited in the supplementary materials.
