# Supplementary material for: Andrographolide Induces ROS-Mediated Cytotoxicity, Lipid Peroxidation, and Compromised Cell Integrity in Saccharomyces cerevisiae
Source: Antioxidants (Basel). 2023 Sep 14;12(9):1765. doi: 10.3390/antiox12091765 (PMC10525756; doi:10.3390/antiox12091765)
Supplement: Supplementary file 1 [file antioxidants-12-01765-s001.zip › antioxidants-2602421-SI.pdf]

Supplementary information

Andrographolide induces ROS-mediated cytotoxicity, lipid peroxidation, and compromised cell integrity in *Saccharomyces cerevisiae*

Tanaporn Phetruen[1], Bloem van Dam[1], Sittinan Chanarat[1,*]

[1]Laboratory of Molecular Cell Biology, Center for Excellence in Protein and Enzyme Technology, Faculty of Science, Mahidol University, Bangkok, Thailand

[*]Correspondence: sittinan.cha@mahidol.edu

Table S1. Strains used in this study

Strain	Genotype	Reference
W303 wild-type	*MAT**a** ho ade2-1 his3-11, 15 leu2-3, 112 ura3 trp1-1 ssd1 can1-100*	K. Nasmyth
sod2Δ	W303 *sod2Δ::kanMX6*	This study
css1Δ	W303 *ccs1Δ::kanMX6*	This study
vma4Δ	W303 *vma4Δ::kanMX6*	This study
vma9Δ	W303 *vma9Δ::kanMX6*	This study
erg6Δ	W303 *erg6Δ::kanMX6*	This study
sGFP-*VRG4*	W303 sGFP-*VRG4*	This study
SEC7-mCherry2Bx6	W303 *SEC7*-mCherry2Bx6	This study
VPS8- mCherry2Bx6	W303 *VPS8*- mCherry2Bx6	This study
ERG11-iGFPx3	W303 *ERG11*-iGFPx3	This study

Table S2. Plasmids used in this study

Plasmid	Description	Reference
YIplac211-sGFP-*VRG4*	Integration plasmid used for fluorescence visualization of early Golgi	(Casler and Glick, 2020)
YIplac211-*SEC7*-mCherry2Bx6	Integration plasmid used for fluorescence visualization of late Golgi	(Casler and Glick, 2020)
YIplac211-*VPS8*-mCherry2Bx6	Integration plasmid used for fluorescence visualization of prevacuolar endosome (PVE)	(Casler and Glick, 2020)
YIplac211-*ERG11*-iGFPx3	Integration plasmid used for fluorescence visualization of endoplasmic reticulum	(Casler and Glick, 2020)

Table S3. Primers used in this study

Primer	Sequence (5'→3')
SOD2-S1	GGAACGAAACCCCAATTGATAACTATACCTCCTAAAAACGTACCAGGATGC GTACGCTGCAGGTCGAC
SOD2-S2	AAAAACGACACAGTGGAAAAAAAAAGGTATTTTCTTTCTTTCTTTCTTCAAT CGATGAATTCGAGCTCG
CCS1-S1	GCACAAAAATGACCACGAACGATACATACGAGGCTACTTATGCCATTCCCA TGCGTACGCTGCAGGTCGAC
CCS1-S2	CTATATTATGTTATATCTGTATTACGCTACGTTGTGCTATCTTGGATGTTCTAA TCGATGAATTCGAGCTCG
VMA4-S1	ACTGTTTTACAAAAGAGGCACAGAACAGGCCACGCACCATCCGATACAGC ATGCGTACGCTGCAGGTCGAC
VMA4-S2	TCTGTTAGGAGTGTATATGTAAGTATGTAGGTATACAAGCTGCTGGTCGATC AATCGATGAATTCGAGCTCG
VMA9-S1	TATTCTGCAAATTCAGGTCTCAAATCTGAACGGCGTGGAGCCACCAAGGG ATGCGTACGCTGCAGGTCGAC
VMA9-S2	TTTTATTCTCGCTCTGTGAAAAAAGACTACTACATTTTTTGACCTTTTATCTA ATCGATGAATTCGAGCTCG
ERG6-S1	AATTTAAAAAAACAAGAATAAAATAATAATATAGTAGGCAGCATAAGATGCGT ACGCTGCAGGTCGAC
ERG6-S2	TAGGTATATATCGTGCGCTTTATTTGAATCTTATTGATCTAGTGAATTTAATCG ATGAATTCGAGCTCG
HAC1-F	CGCAATCGAACTTGGCTATCCCTACC
HAC1-R	CCCACCAACAGCGATAATAACGAG

Figure S1

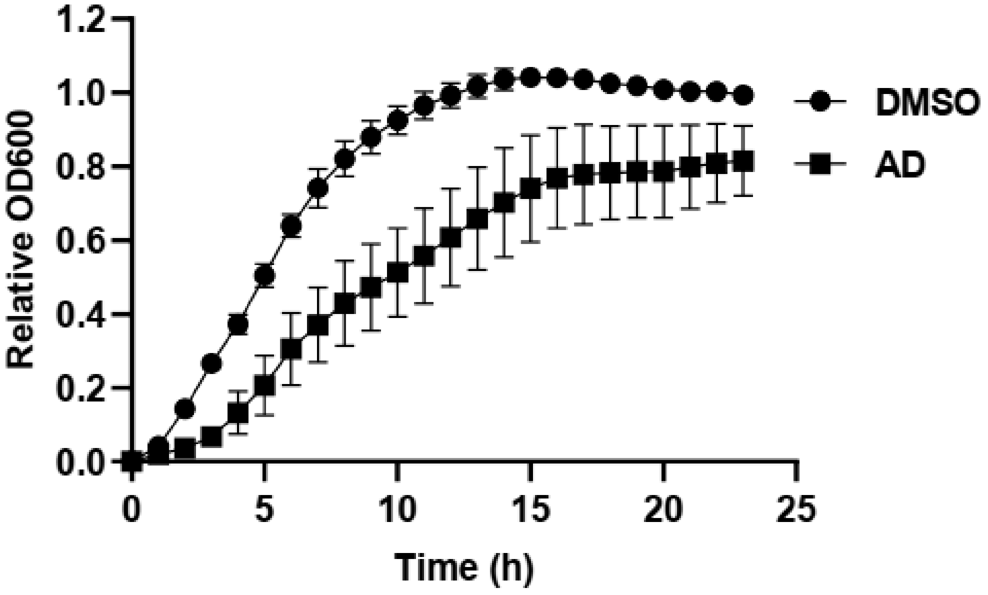

Supplementary Figure S1. Andrographolide exhibits cytotoxicity in *S. cerevisiae* with ROS synergy. Similar to Figure 1A, but wild-type yeast cells were grown in synthetic minimal media supplemented with 2% glucose.

Figure S2

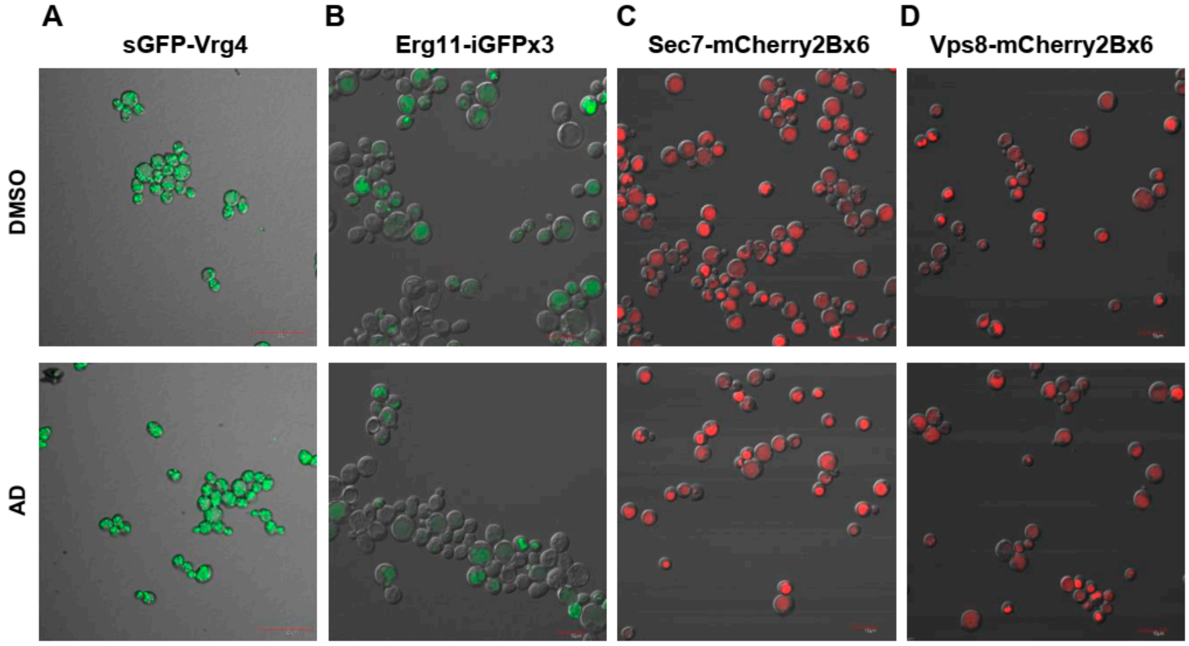

Supplementary Figure S2. No significant change in morphological abnormalities of organelles was observed in cells treated with andrographolide. (A) – (D) Fluorescence micrograph of cells expressing sGFP-Vrg4, Sec7-mCherry2Bx6, Vps8-mCherry2Bx6, and Erg11-iGFPx3, respectively. Cells were treated with or without andrographolide. DMSO serves as solvent control. Scale bar, 10 µm.

Figure S3

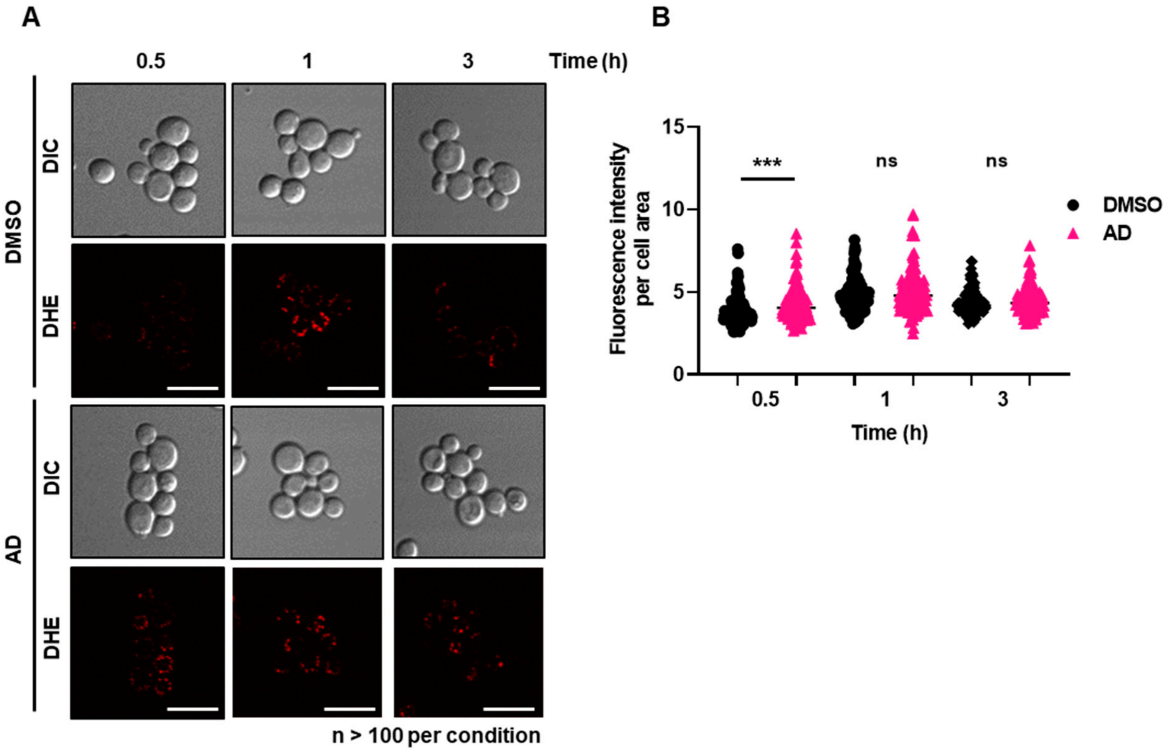

Supplementary Figure S3. Levels of superoxide remained unchanged upon andrographolide treatment. (A) DMSO- or andrographolide-treated cells were assayed with DHE for quantification of superoxide levels in cells. Scale bar, 10 □m. (B) Quantification of Figure S3A. Each dot represents fluorescence intensity per cell area measured by ImageJ. Statistical significance was calculated using two-tailed unpaired t-test with Mann-Whitney test (***, $p < 0.001$; ns, not significant).

Figure S4

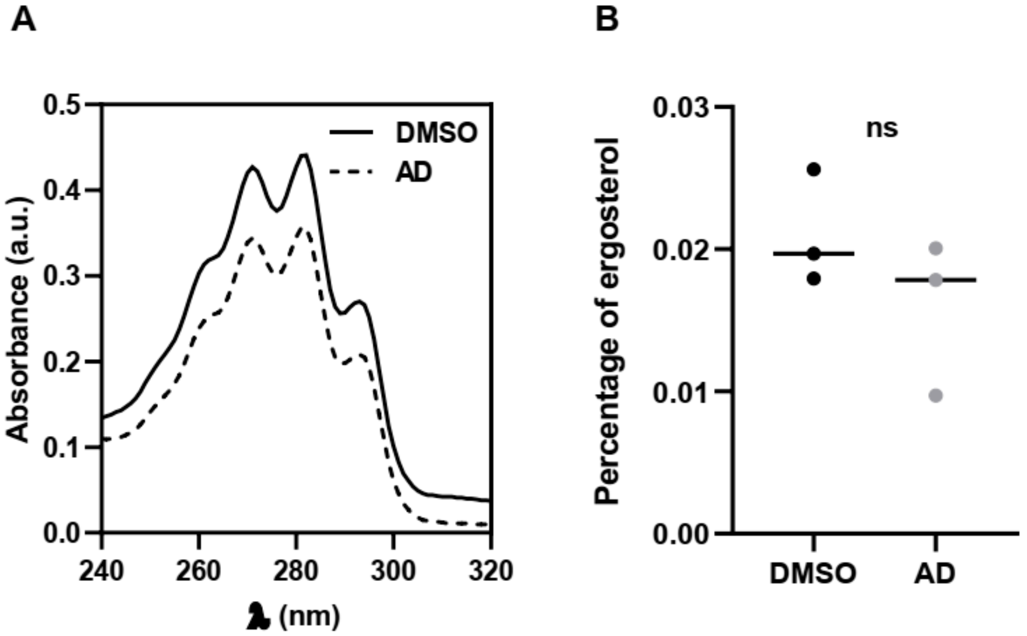

Supplementary Figure S4. Levels of ergosterol remained unchanged upon andrographolide treatment. (A) Sterols were extracted from the cells treated with or without andrographolide and spectrophotometrically scanned from 240 to 320 nm. The characteristic four peaks of sterols were shown. (B) Ergosterol contents were calculated from values obtained from Figure S4A and plotted. Each dot represents the percentage of ergosterol from three biological replicates. Statistical analysis was carried out using a two-tailed unpaired t-test with Mann-Whitney test (ns, not significant).

Figure S5

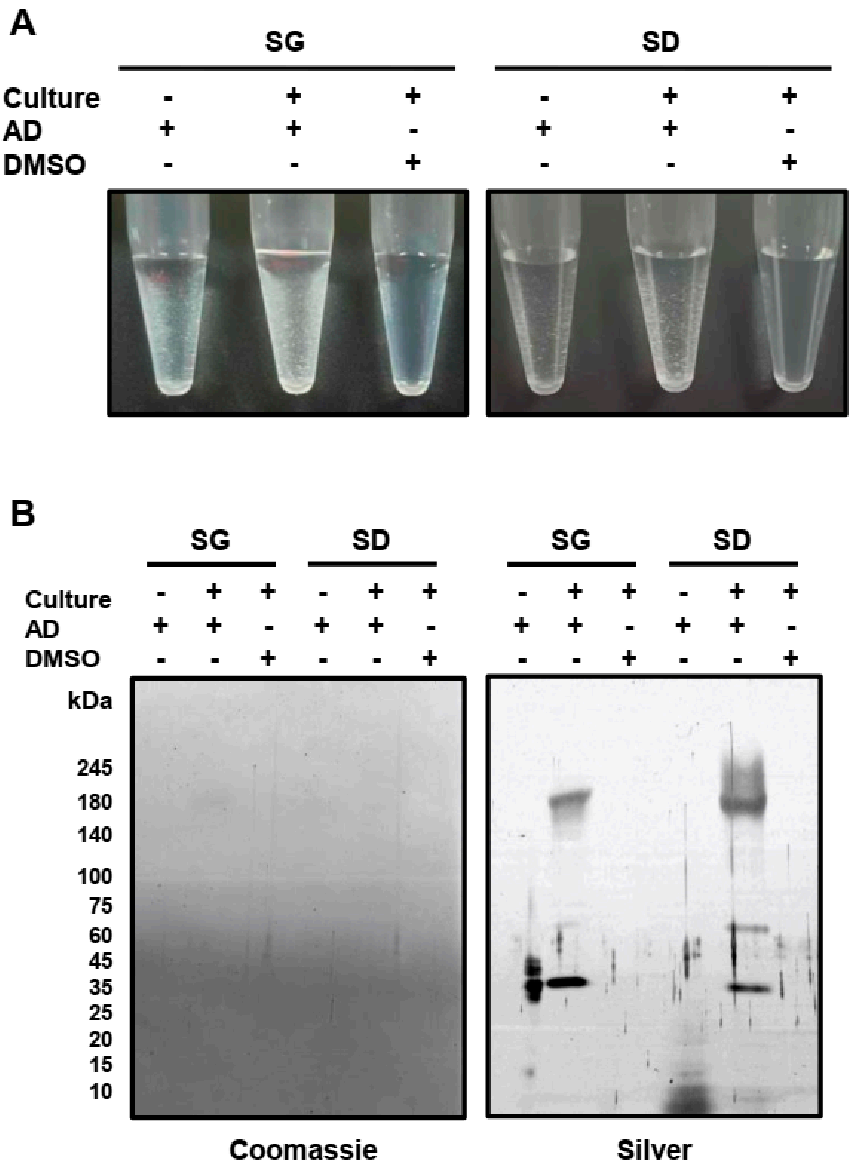

Supplementary Figure S5. Andrographolide increases precipitation in cultured media. (A) The precipitation in SG and SD media from the experiment shown in Figure 6B. (B) The pellets were resuspended in 2x SDS-PAGE loading buffer and subjected to SDS gel electrophoresis followed by Coomassie blue and silver staining.

Supplementary reference

Casler, J.C., and Glick, B.S. (2020). A microscopy-based kinetic analysis of yeast vacuolar protein sorting. Elife *9*.
